# Supplementary material for: Technical efficiency of primary health care facilities in providing adolescent mental, sexual and reproductive health services in Ghana: A case study of selected districts in the Greater Accra Region
Source: PLoS One. 2025 Jun 3;20(6):e0321265. doi: 10.1371/journal.pone.0321265 (PMC12133010; doi:10.1371/journal.pone.0321265)
Supplement: Attachment — Submitted filename: PONE-D-24-09524_Review.pdf [file pone.0321265.s002.pdf]

Summary

This study estimates the technical efficiency of adolescent sexual and reproductive health services among healthcare facilities in Greater Accra Region, Ghana and their environmental determinants. The authors use stochastic frontier analysis to estimate the technical efficiency of each healthcare facility, then multivariable regression analysis to assess the statistical relationships between environmental characteristics and technical efficiency. The authors find that healthcare facilities provide adolescent sexual and reproductive health services at approximately 60% efficiency, but with significant heterogeneity across the sample. Services covered by clinical staff, insurance coverage, the Gini Index, and the literacy rate were all statistically significant. This research provides an assessment of technical efficiency of specific services that are vital for the growing youth population in Ghana, as well as evidence for future policies that could improve the efficiency of those services if implemented.

General feedback

- Make sure acronyms are clearly defined the first time they are used in writing. There are several acronyms that are not defined.
- If there are no mental health services included in the analysis, then the title and writing within the article should change to reflect this.

Materials and methods

- Lines 136-138: Please clarify the data source for this study. In Line 137, it says the data was accessed, so it is unclear whether this is primary or secondary data.
- Lines 136-138: Please provide more information on how facilities were contacted and enrolled. Do you have an idea of the total number of AMSRH facilities there are in each district or Accra?
- Line 141: Please provide more information on how you determined the sample size. You described the different levels and clusters, but you have not stated the number of facilities you need for your analysis.
- Lines 150-152: What were common reasons for excluding facilities from the analysis?
- Line 183: Can you provide some examples of studies that have used the Cobb-Douglas and Translog functions as references?
- Lines 199-204: Please provide references to justify the decision to orient the model toward outputs.
- Lines 206-209: Please provide some justification for the variables you include in the model. Are these variables similar to the ones included in analogous studies?
- Line 208: In Table 1, please clarify the unit of medicines or medications, such as number of doses instead.
- Line 208: For Table 1, I recommend organizing the variables by socioecological level. For example, clinic-level variables should be listed first, followed by district-level variables.

- Lines 218 and 223: Can you clarify the meaning behind service intensity?

Results

- Line 234: For all variables in Table 2, please provide the unit for each variable.
- Line 234: There are many variables that are not normally distributed. Have you checked whether skewness impacts the distribution of residuals and the estimation of your models?
- Line 246-247: Can you explain how the estimated parameters are acceptable for the model?
- Line 252: In Table 3, please define the metric in parentheses. Is it the standard errors for regression coefficients?
- Line 252: In Table 3, please identify which summary statistics are means or proportions. Please consider omitting the range for those variables that are proportions. (For example, the range of urban facility location is from 0 to 1.)
- Lines 256-266: Can you interpret the efficiency scores by facility location, type, etc. as being statistically different from each other, considering the means cross into the 95% confidence intervals, as described in Table 4?
- Line 269-270: Please rewrite the sentence "A positive (negative) estimate…" to be clearer.
- Lines 274-275: This is not a complete sentence.
- Lines 269-277: The model for the results described in Table 5 is not presented in the methods section. Please provide some description of the model there.

Discussion

- Lines 290-295: These four sentences essentially say the same thing. Consider condensing these four sentences into fewer.
- Lines 295-296: Are these findings in line with similar research, either on other healthcare services in Ghana or facilities in other African countries?
- Lines 299-311: You suggest that these services could increase their efficiency by providing more resources, yet the solutions you identify pertain to greater utilization by the patient population. Please provide some examples of these resources.
- Lines 303-307: These qualitative findings need more context. Did you interview potential patients? Were these findings relevant across types of facilities?
- Lines 323-331: You discuss the role of insurance in increasing healthcare utilization. How does the insurance rate in the district, though, improve the technical efficiency of facilities? Could it be because it makes healthcare administration run faster? Their services better match the needs of the patient population? Please discuss this, especially since it is the only variables that were positively associated with technical efficiency.
- Can you speak to why the literacy rate is negatively associated with technical efficiency? This seems counterintuitive.
- Lines 338-340: What characteristics might limit the generalizability of the findings? Higher poverty in other regions? Lower healthcare provider density?

Conclusion

- Lines 350-352: I am not sure there is adequate evidence for the roles of patient privacy or the scope of services in facility efficiency. I only recall patient privacy mentioned in the discussion as anecdotes that were outside of the main analysis. As for the scope of services, there was only a small statistically significant association between the number (not scope) of services and efficiency.
