# Supplementary material for: Technical efficiency of primary health care facilities in providing adolescent mental, sexual and reproductive health services in Ghana: A case study of selected districts in the Greater Accra Region
Source: PLoS One. 2025 Jun 3;20(6):e0321265. doi: 10.1371/journal.pone.0321265 (PMC12133010; doi:10.1371/journal.pone.0321265)
Supplement: Attachment — Submitted filename: Response to Reviewers.docx [file pone.0321265.s004.docx]

30^th^ October, 2024

To: Editor-in-Chief

PLOS One

Dear Editor,

I am resubmitting a revised copy of the manuscript below to be considered for your journal:

**Technical Efficiency of Primary Health Facilities in Providing Adolescent Mental, Sexual and Reproductive Health Services in Ghana: a case study of selected districts in the Greater Accra Region**

Jacob Novignon, Ama Pokuaa Fenny, Michel Adurayi Amenah, Selassie Adom, Annick Gladzah, Nassirou Ibrahim, Ludovic Deo Gracias Tapsoba, Irene A. Agyepong, Roxanne Borges da Silva6, Tim Ensor

We are grateful for the comments and suggestions from our reviewers. We have addressed and responded to each reviewer in the text below.

Yours sincerely,

Ama Pokuaa Fenny, PhD

**Response to Reviewers**

**Manuscript Title**: Technical Efficiency of Primary Health Care Facilities in Providing Adolescent Mental, Sexual and Reproductive Health Services in Ghana: A Case Study

**Manuscript ID**: PONE-D-24-09524

**Reviewer #1:**

1. **Title Revision**

**Comment**: "Title needs to be more concise and understandable. It is better to use 'Primary Health Care Facilities.'"

**Response**: We have revised the title to: *"Technical Efficiency of Primary Health Care Facilities in Providing Adolescent Mental, Sexual and Reproductive Health Services in Ghana: A Case Study"*, in line with your suggestion"

2. **Abstract Revision**

**Comment**: "The abstract is too long. It is better to shorten the background."

**Response**: Many thanks for the comment. We have now revised the entire abstract to make it shorter while keeping essential information.

3. **Introduction Revisions**

**Comment**: "The introduction is too large. Avoid providing similar information in several sentences."

**Response**: The introduction has been condensed, and similar points were combined into single sentences with additional references, as requested.

4. **Methodology Details**

**Comment**: "More details are needed in the methodology, such as how many facilities were selected, urban vs. rural split, and consent process."

**Response**: We have provided more detailed information in the methodology section to address the reviewer’s concerns. Specifically, we clarified that all primary healthcare (PHC) facilities in the four selected districts were included, meaning we did not specifically select public or private facilities but rather covered all available PHC facilities in these districts. In addition, we included the consent process for data collection.

5. **Result Revisions**

**Comment**: "p-value needed in Table 2, and breakdown of services using Likert scale."

**Response**: We appreciate the comment however in Table 2 we simply present summary statistics of the key variables. The summary statistics reports just the means and standard deviation of the variables. For univariate analysis of this nature, p-values are not reported. We do not have a variable that measures service using the Likert scale.

6. **Conclusion Revisions**

**Comment**: "The conclusion is too generic. It should be improved to reflect the specific findings."

**Response**: We appreciate the reviewer’s feedback and agree that the initial conclusion did not fully reflect the specific findings of the study. We have revised the conclusion to better emphasize the key results, including the observed inefficiencies in resource use for adolescent sexual and reproductive health (ASRH) services and the minimal provision of adolescent mental health services. We now highlight specific aspects such as the need for patient privacy, broader service provision, and improvements in environmental factors like financial access to healthcare.

**Reviewer #2**

1. **Introduction Refocus**

**Comment**: "Refocus the introduction around resource limitations and the need to maximize AMSRH resources given the demand/burden of disease."

**Response**: We agree with the reviewer on this. Indeed, a similar comment was also made by other reviewers. We have therefore revised the entire introduction accordingly, with a focus on resource limitations and the need to maximize adolescent health resources.

2. **Define Inputs and Outputs in Layman's Terms**

**Comment**: "Define inputs and outputs earlier in the methods and use them to define the analysis in layman’s terms."

**Response**: Thank you for this important comment. In response to your suggestion, we have included more details to table 1 to make the variables more relatable. This table presents the variables in a structured and easy-to-understand format to enhance clarity for the reader.

3. **Methodology Adjustments**

**Comment**: "Do not discuss the paucity of mental health care in Methods; that is for the Results."

**Response**: Thank you. We have now removed the discussion of the paucity of mental health care from the methodology and included it in the results section.

4. **Discussion Revisions**

**Comment**: "Start the discussion with a summary of the results, then discuss the broader impact on resource efficiency in low-resource settings."

**Response**: The discussion now begins with a summary of the results, followed by an exploration of how the findings can be applied to improve technical efficiency in low-resource settings. Specific recommendations are made for prioritizing efforts to improve resource efficiency.

5. **Privacy in Discussion**

**Comment**: "De-emphasize privacy in the discussion unless you have additional data on privacy to contribute."

**Response**: We de-emphasized the focus on privacy in the discussion, as we do not have additional data to support its relevance in this context.

**Reviewer #3:**

1. **Technical Efficiency Estimation**
   - **Comment**: "This study estimates technical efficiency of adolescent health services using stochastic frontier analysis."
   - **Response**: We appreciate the recognition of the value of our methods. No further changes were necessary in this regard.

**Additional Revisions**

1. **Data Availability**
   - **Comment**: "Ensure all data underlying the findings are fully available."
   - **Response**: We have uploaded the necessary raw data (including values behind means, standard deviations, and other measures) as Supporting Information files. The minimal dataset required to replicate our findings is now fully available.
2. **Language and Grammar**
   - **Comment**: "Thoroughly copyedit the manuscript for grammar and spelling."
   - **Response**: The manuscript has been reviewed and copyedited for language consistency, grammar, and clarity.
3. **Ethics Statement**
   - **Comment**: "Include full ethics statement in the Methods section."
   - **Response**: The ethics statement has been expanded to include details about the IRB approvals, informed consent from facility heads, and the anonymity of collected data.

### **Other comments**

### **Materials and Methods**

1. **Lines 136-138: Clarify data source**

**Response:** The data for this study is primary data collected from primary health facilities in four districts of the Greater Accra Region. This has been clearly stated in the revised manuscript.

2. **Lines 136-138: Provide information on how facilities were contacted and enrolled**

**Response:** The facilities were contacted through the District Health Directorate in each district, which provided a list of all functional primary healthcare facilities. The directorate also supplied the addresses and contact information of the heads of each health facility.

3. **Line 141: Provide information on sample size determination**

**Response:** . For each district, data was collected from all primary healthcare facilities. However, only facilities with complete data were included in the final analysis. Facilities with missing information on key input and output variables were excluded, resulting in a final sample of 53 facilities.

4. **Lines 150-152: Common reasons for excluding facilities**

**Response:** Sick bays (or school clinics) were excluded from the analysis as they do not provide Adolescent Sexual and Reproductive Health (AMSRH) services.

5. **Line 183: Examples of studies using Cobb-Douglas and Translog functions**

**Response:** Examples from the literature where the Cobb-Douglas and Translog functions are used and has been added to provide context and justification. See “Colther C, Doussoulin JP. Recent Applications and Developments of the Cobb–Douglas Function: From Productivity to Sustainability. Journal of the Knowledge Economy. 2024 May 16; Subal C. Kumbhakar, Hung-Jen Wang. Estimation of Technical Inefficiency in Production Frontier Models Using Cross-Sectional Data. Indian Econ Rev. 2010 Dec; Vol. 45(No.2):7–77.”

6. **Lines 199-204: Justification for output orientation**

**Response:** In this study, we used the output orientation given that the health facilities only receive a set of resources and the decision under their control is usually how much outputs can be produced with the available resources. Moreover, the ultimate objective of health facilities is to expand output rather than limiting available resources. We have made this point in the revised manuscript.

7. **Lines 206-209: Justification for variables**

**Response:** The variables used in this study are consistent with those found in analogous studies assessing health facility efficiency. The Stochastic Frontier Analysis (SFA) approach begins with estimating production functions that establish the relationship between inputs and outputs. In this study, AMSRH outputs are captured as outpatient attendance in a particular year. The key inputs necessary to produce these outputs include the health workforce and the supplies required to provide the services. This alignment of variables with established literature supports the validity of efficiency assessment.

8. **Line 208: Clarification of the unit for medicines**

**Response:** The term "medicines" has been clarified as the "number of packs" of medicines disbursed rather than doses, and this clarification has been added in Table 1.

9. **Line 208: Organize variables by socioecological level**

**Response:** We considered the reviewer’s suggestion to organize the environmental variables into categories such as health facility environment, community, and district. However, we argue that presenting the variables in their current form, categorized as inputs, outputs, and environmental factors provides a clearer understanding of their role in efficiency analysis. This structure aligns with the logic of the Stochastic Frontier Analysis (SFA) framework, where inputs are used to produce outputs, while environmental factors represent external conditions affecting efficiency. A brief explanation of this rationale has been added to Table 1 for clarity.

10. **Lines 218 and 223: Clarify the meaning of service intensity**

**Response:** Service intensity refers to the variation in the complexity or severity of cases managed at a health facility. Facilities handling more severe or complex cases require more resources. This has been clarified in the revised manuscript.

**Results**

11. **Line 234: Provide the unit for each variable in Table 2**

**Response:** Units for each variable (e.g., time in minutes, medicines in packs, etc.) has been added to Table 2.

12. **Line 234: Skewness and its impact on the distribution of residuals**

**Response:** Thank you for this comment. The stochastic frontier analysis does impose distribution assumption on the error term. The validity of this assumption is tested by the value of lampda as discussed in the manuscript.

13. **Line 246-247: Explain how estimated parameters are acceptable**

**Response:** The value of lambda, as reported in Table 3, indicates the presence of inefficiency in the data, which is crucial for estimating the SFA model. The statistically significant coefficient further confirms the existence of inefficiency in the dataset.

14. **Line 252: Define the metric in parentheses (Table 3)**

**Response:** The values in parentheses in Table 3 represent the standard errors of the regression coefficients.

15. **Line 252: Identify means or proportions in Table 3**

**Response:** Table 3 reports coefficients and standard errors from the estimation of the production function

16. **Lines 256-266: Statistically different efficiency scores**

**Response:** Thank you for the observation. In Table 4, all the differences in efficiency reported across facility characteristics were statistically different from zero or statistically significant. We have indicated this in the manuscript.

17. **Line 269-270: Clarify sentence about positive/negative estimate**

**Response:** Table 5 presents the regression results on the environmental determinants of technical efficiency. A positive estimate indicates a factor that improves efficiency, while a negative estimate indicates a factor that reduces efficiency. This has been clarified in the revised manuscript.

18. **Lines 274-275: Incomplete sentence**

**Response:** The sentence has been completed and rewritten for clarity.

19. **Lines 269-277: Describe the model used in Table 5**

**Response:** The Tobit model was used to produce the estimates in Table 5. We have included a short section in the methods explaining the model.

**Discussion**

20. **Lines 290-295: Condense sentences**

**Response:** These sentences has been condensed to eliminate redundancy while maintaining clarity.

21. **Lines 295-296: Compare findings with similar research**

**Response:** The findings of this study have been compared with similar research on healthcare services in Ghana and other African countries. For instance, inefficiencies in healthcare have been reported in studies conducted in comparable settings. However, it is important to note that this is the first study of its kind to estimate efficiency in the use of resources specifically for AMSRH services. As a result, there is limited previous research directly related to AMSRH to compare with. Nonetheless, we have included research on efficiency in other specific programs or disease areas, which are also relatively limited. This comparison provides a broader context for interpreting our results while highlighting the unique contribution of our study.

22. **Lines 299-311: Provide examples of resources**

**Response:** Examples of resources that could improve efficiency include increasing the number of clinical staff, improving facility infrastructure, and providing more diagnostic equipment. These examples have been included in the revised manuscript. However, simply increasing the number of clinical staff will not necessarily improve efficiency. Ensuring that staff are appropriately allocated is crucial. For instance, dispatching clinical staff to facilities where they are already in abundance could worsen efficiency, whereas sending them to facilities facing staff shortages would likely enhance efficiency. This targeted allocation of resources is essential for improving overall healthcare efficiency. See from lines 391-397.

23. **Lines 303-307: Context for qualitative findings**

**Response:** We did not mention qualitative findings in the manuscript. Instead, we referred to “observations from the fieldwork,” such as noting that adolescents may find it difficult to seek care at the facilities due to the sensitive nature of sexual and reproductive health services. These observations were informal and anecdotal, gathered during interactions with healthcare workers rather than through a formal qualitative method. We have ensured that this distinction is clearly reflected in the revised manuscript, replacing any mention of formal findings with “observations during fieldwork” as appropriate. See line 402.

24. **Lines 323-331: Explain how insurance improves efficiency**

**Response:** . Insurance improves efficiency by reducing financial barriers to care, which increases healthcare utilization and enables health facilities to optimize their services to meet patient needs. In the context of our study, the outcome is outpatient visits, which partly depends on the ability of adolescents to seek care. Where insurance coverage exists, adolescents are more likely to seek care as financial barriers are removed. This leads to an increase in outpatient visits, allowing for better use of facility resources and reducing redundancy in inputs. This has been discussed in greater detail in the revised manuscript.

25. **Explain why literacy rate is negatively associated with technical efficiency**

**Response:** The results regarding literacy were unexpected and contrary to our initial expectations. We had anticipated, at most, an insignificant association. However, the observed negative association provides a basis for further investigation into this relationship. This has been noted in the discussion section for further exploration.

26. **Lines 338-340: Generalizability limitations**

**Response:** . The generalizability of the findings may be limited by factors such as higher poverty rates, lower healthcare provider density, and fewer healthcare resources in other regions, which have been discussed in the revised manuscript. Our findings are only generalizable in contexts where primary healthcare facilities are designed to provide comprehensive AMSRH services. In Ghana, given the similar setup of primary healthcare facilities across the country, our findings can be generalized within the national context. However, generalizing these results to other countries should be done with caution, considering differences in healthcare systems and resource availability.

**Conclusion**

27. **Lines 350-352: Patient privacy and scope of services**

**Response:** The discussion of patient privacy and scope of services has been toned down as there is insufficient evidence from the study to make strong conclusions on these aspects. Instead, the focus is on the scope and breadth of services and how they can improve efficiency. See from line 456 - 464
