# Supplementary material for: Technical efficiency of primary health care facilities in providing adolescent mental, sexual and reproductive health services in Ghana: A case study of selected districts in the Greater Accra Region
Source: PLoS One. 2025 Jun 3;20(6):e0321265. doi: 10.1371/journal.pone.0321265 (PMC12133010; doi:10.1371/journal.pone.0321265)
Supplement: Attachment — Submitted filename: Response_to_Reviewers_auresp_2.docx [file pone.0321265.s005.docx]

**Response to Reviewers**

Dear Academic Editor,

We appreciate the opportunity to revise and resubmit our manuscript titled "Technical Efficiency of Primary Health Facilities in Providing Adolescent Mental, Sexual and Reproductive Health Services in Ghana: A Case Study of Selected Districts in the Greater Accra Region" (PONE-D-24-09524R1). We thank you and the reviewers for your valuable feedback, which has helped us improve the clarity and quality of our manuscript. Below, we provide a point-by-point response to each of the comments raised by the editor and reviewer(s).

**Editor's Comments**

| **Comments** | **Response** |
| --- | --- |
| In your abstract section, "Primary healthcare (PHC) facilities have become essential in promoting adolescent healthcare, yet they face resource limitations that hinder their effectiveness. Ensuring the efficient use of available resources has therefore become pertinent.” Yes, it is important, but what was your research questions?  Describing the research question (gaps) that led you to assess the technical efficiency of primary health care facilities benefits the clarity before you state your objective (i.e., This study assessed.....) | Thank you for this observation. We added the research question in the abstract in the following sentence. “This study assessed the technical efficiency of primary health care facilities in providing adolescent mental sexual and reproductive health (AMSRH) services” |
| Numbering headings, and sub-heading adds minimal benefits to the clarity of your content in some sections. So, it’s important to think over removing numbering of headings and subheadings  or  collapsing some third-level headings into second level, and succinctly describing them benefit the clarity and organization of the manuscript. e.g. collapse "2.3.1 "  in your method section into  2.3, and do the same thing in your result section, eg. 3.2.1.... Finally, avoid the sub-sectioning, and continue as “Introduction”, “methods”, “results”, Discussion”, “conclusion”... | This is well noted. We have accordingly merged some of the subheadings. We have also removed all of the sub sectioning in the results section and kept only essential subheadings in the methods section. |
| **Results Section:** "Break your '3.0 Results and Discussion' into separate sections: 'Results' and 'Discussion'." | We have done exactly as suggested. |
| Your discussion seems to lack depth. Please first, summarize what you learned from the results, and discuss and compare your findings with other studies in Ghana. There are studies within Ghana, and other countries with nearly similar contexts,  possible bias, limitations, policy implications etc | Thank you for this comment. The discussion includes these themes. We sumarize the findings in the first paragraph and compre them to previous studies. We note that while previous studies have studied general healths ervice delivery, our findings are peculiar to ASRH service delivery and this limits the extent of comparison. We highlight the intersection of our findings with policy and outline the strengths and limitations. |

**Reviewer's Comments (Reviewer #3)**

| **Comments** | **Response** |
| --- | --- |
| In the introduction, please carefully review the formatting for citations, since several are outside of the sentences to which they belong. | The formatting for citations in the introduction has been carefully reviewed and corrected to ensure that all references are appropriately placed within the relevant sentences. |
| In the materials and methods section, please define DEA (line 126) and add some context to what "flexibility" means for the translog function (line 151). | Thank you for the obseervation. We have now defined DEA as data envelopment analysis. The term flexibility in this context refers to the model’s ability to allow cross-product terms of the input variables. This accounts for complementary and substitution effects in the model. We have now made this clearer in the text. |
| For the results, in Table 2, please clarify what "Insurance" as a characteristic means. Also, I want to reiterate that, for lines 262-273, for many of the facility characteristics, the differences in mean efficiency scores is very small and the confidence intervals overlap. For example, the mean score for rural facilities is 0.630, but that score is fits in the confidence interval for urban facilities. Therefore, I am unsure if you can say that they differences are statistically significant, as you do in lines 272-273. This is even more pronounced--that the mean scores are very narrowly different--for characteristics like facility laboratory or gender of facility head. | Many thanks for this important observation. We have now clarrified that the variable captures health insurance coverage in the district.  The Cis reported in Table 4 do not overlap as suggested in the comment. We claim statistical significance because none of the CI ranges include the null value. We also did acknowledge that for some of the characterisitcs the mean difference were small. |
| Finally, for the discussion, I saw that you revised the writing around fieldwork observations. I suggest adding some language to couch the statements as from your or the data collection team's perspectives. For example, "We heard from adolescent patients that they would prefer to self-medicate..." While I saw that you addressed my comment about sample size, I recommend that you discuss this as a limitation to the study. I am not familiar with the SFA, but I wonder whether ~50 firms in the analytic sample is sufficient, especially with the 20 covariates in the final models. I looked around and found some studies that simulate SFA models under different sample sizes, like Cheng et al. (2024): <https://www.sciencedirect.com/science/article/pii/S0304407622000677> | Thank you for the observation. We have clarrified that we heard from health workers who have substantial knowledge about health seeking behavious.  With regards to the sample size, we agree that larger sample sizes help model predictability as is ths case in any regression model. However, for 20 variable model, a sample of 50 is enough. It is also worth mentioning that this paper is based on four districts and we included all primary health facilities in these districts. Also thank you for sharing the paper by Cheng et al (2024) even though they only deal with distributional assumptions of the inefficiency term. |
